# Myoelectric Signal Classification of Targeted Muscles Using Dictionary Learning

**DOI:** 10.3390/s19102370

**Published:** 2019-05-23

**Authors:** Hyun-Joon Yoo, Hyeong-jun Park, Boreom Lee

**Affiliations:** Department of Biomedical Science and Engineering (BMSE), Institute of Integrated Technology (IIT), Gwangju Institute of Science and Technology (GIST), Gwangju 61005, Korea; pmnrmyoo@gist.ac.kr (H.-J.Y.); phyeong106@gist.ac.kr (H.-j.P.)

**Keywords:** electrodes, electromyography, prosthetic hand, myoelectric control, dictionary learning

## Abstract

Surface electromyography (sEMG) signals comprise electrophysiological information related to muscle activity. As this signal is easy to record, it is utilized to control several myoelectric prostheses devices. Several studies have been conducted to process sEMG signals more efficiently. However, research on optimal algorithms and electrode placements for the processing of sEMG signals is still inconclusive. In addition, very few studies have focused on minimizing the number of electrodes. In this study, we investigated the most effective method for myoelectric signal classification with a small number of electrodes. A total of 23 subjects participated in the study, and the sEMG data of 14 different hand movements of the subjects were acquired from targeted muscles and untargeted muscles. Furthermore, the study compared the classification accuracy of the sEMG data using discriminative feature-oriented dictionary learning (DFDL) and other conventional classifiers. DFDL demonstrated the highest classification accuracy among the classifiers, and its higher quality performance became more apparent as the number of channels decreased. The targeted method was superior to the untargeted method, particularly when classifying sEMG signals with DFDL. Therefore, it was concluded that the combination of the targeted method and the DFDL algorithm could classify myoelectric signals more effectively with a minimal number of channels.

## 1. Introduction

Upper limb amputations cause severe functional disability and significantly affect the daily lives of patients. A recent study showed that more than 30,000 patients undergo upper limb amputation surgery in the United States annually due to trauma or vascular diseases [1]. Unfortunately, most amputees are still young, and it is essential to make an effort to restore their lost functions so that they can return to daily life. As a part of this effort, research on hand transplantation is underway; however, several problems must be resolved before this treatment can be applied widely, such as immunosuppressive complications and financial burdens [2]. For this reason, a primary feasible treatment option for upper limb amputation is prostheses, which are used by approximately 80% of patients in daily life [3]. Several types of upper limb prostheses have been developed, which can be classified into four categories: passive prostheses, body-powered prostheses, externally powered prostheses, and myoelectric prostheses. The passive prostheses are the most favorable, because they are low cost and easy to apply. However, they are only intended for cosmetic purposes and do not provide any functional movements [4]. To overcome these limitations, myoelectric hand prostheses are currently being studied extensively. These prostheses are more functional than the passive prostheses and are more intuitive and feasible than body-powered and externally powered prostheses.

Surface electromyography (sEMG) signals are electrophysiological signals that consist of motor unit action potentials propagating along with the skeletal muscles [5]. Such signals are easily recorded on the surface of the skin overlying the muscles and contain information regarding their specific movements. Owing to these properties, sEMG patterns can be analyzed to classify a variety of movements [6]. Consequently, researchers are attempting to develop myoelectric hand prostheses that utilize the principle of myoelectric pattern recognition. However, these products are limited to performing only a few simple hand movements, such as power grip and lateral grip [7].

To resolve this problem, many studies have been conducted on pattern recognition systems to classify more hand gestures with greater accuracy. Most of the previous studies focused on the development of effective processing algorithms for classification. Although there is insufficient consensus on optimal classification and feature sets, time-domain features classified with linear discriminant analysis (LDA) and time-frequency domain features with support vector machine (SVM) have been suggested as optimal algorithms for sEMG classification [8,9]. In addition, approaches, such as the heuristic fuzzy logic classifier [10], Gaussian mixture model [11], and hidden Markov model [12], have been investigated. EMG pattern recognition using high-density sEMG signals is also underway [13,14]. However, although the classification accuracy can be increased by using high-density sEMG sensors, the equipment becomes complicated, and huge computational power is required to process all the sEMG signals in real time. Furthermore, it is difficult to locate several sEMG sensors on the residual muscle of the amputee. Thus, it is desirable to minimize the number of sensors to enhance the usability of sEMG systems. This will reduce the cost and complexity of the hardware. However, well-organized studies focused on reducing the number of channels while maintaining relatively good performance are quite scarce. Placing electrodes on specific muscles seems to acquire more accurate signals, and this might contribute to reducing the number of channels. However, previous studies have shown that targeting electrodes using surface or intramuscular electrodes do not outweigh the untargeted approach [15,16].

Recently, many researchers have applied sparse representation-based methods to classify various features, such as facial recognition [17,18] and image classification [19,20,21]. Furthermore, sparse representation and dictionary learning methods have been used recently to classify various biosignals. Shin et al. proposed a sparse representation classification (SRC) method to distinguish right and left hand movements using electroencephalogram signals [22]. They employed common spatial pattern features as the training dataset and then constructed these features into a dictionary. Zhou et al. proposed a discriminative dictionary learning method for brain–computer interface applications [23]. They added a discriminative term to the k-singular value decomposition (K-SVD) dictionary learning algorithm for the optimization of the dictionary. Rasool et al. also proposed a new dictionary learning method for the classification of electroencephalogram (EEG) signals [24]. They compared a label consistent K-SVD (LC-KSVD) algorithm with their proposed method and suggested that the proposed method showed better performance. Tong et al. proposed dictionary learning for vector quantization feature extraction in electrocardiography by employing k-medoids clustering to avoid the interference of noisy data, which showed better results than other features [25]. As discussed above, the dictionary learning method has demonstrated excellent results for the classification of biosignals. However, the dictionary learning method has not yet been applied to sEMG, to the best of our knowledge. Discriminative feature-oriented dictionary learning (DFDL), designed to classify histopathological images, performed well in cases where the training set size was limited [20]. According to previous studies, experiments conducted to classify hand gestures comprised less than 10 trials for each hand gesture [15,16,26]. In this study, we propose a framework to classify sEMG signals using DFDL.

The aim of this study was to investigate the efficacy of applying DFDL in a myoelectric prosthetic hand control system. An offline study was conducted to examine the characteristics of the algorithm under controlled laboratory conditions. In particular, we tried to determine the effect of DFDL on the robustness of pattern recognition using a small number of channels. Subsequently, we studied the effects of placing electrodes on targeted muscles. Unlike previous studies, we assumed that placing electrodes on the targeted physiologically appropriate muscles would be more intuitive and perform better than placing electrodes without targets. To summarize, we hypothesized that placing electrodes on targeted muscles and analyzing sEMG signals with DFDL would be an effective method to classify pattern recognition. We expected that combining two methods would help to minimize the number of channels.

## 2. Methods

### 2.1. Subjects

A total of 23 healthy subjects aged 21–30 years (17 males and 6 females; 22 right-handed and 1 left-handed) were recruited in this study. None of the subjects had any muscular or neurological disorders. Prior to the experiment, the subjects were instructed to perform a pre-test of 10 min to familiarize themselves with the experimental protocol. The Institutional Review Board of the Gwangju Institute of Science and Technology approved all the procedures and protocols in the study (20180316-HR-34-02-02). All the participants were then asked to read the experiment’s introduction and give their written informed consent.

### 2.2. Electrode Configuration

**Figure 1:**
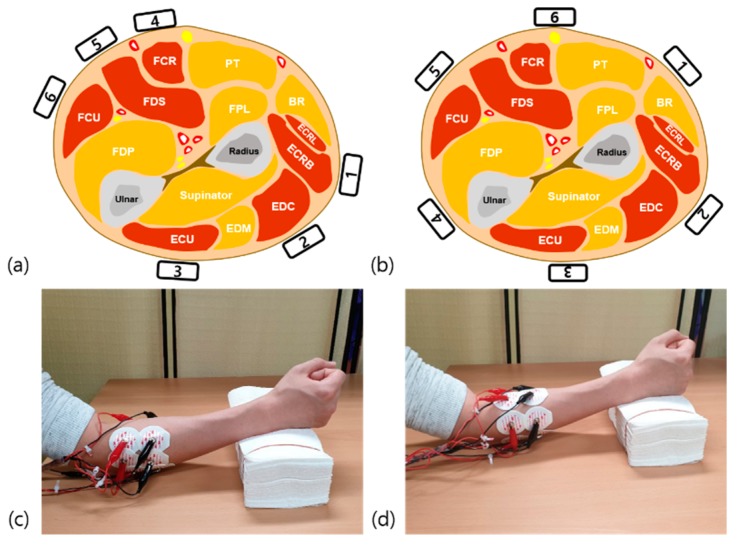
Location of electrodes according to each method. (**a**) Cross section of the right forearm muscle and location of six targeted electrodes. (**b**) Location of six untargeted electrodes. BR: brachioradialis; ECRB: extensor carpi radialis brevis; ECRL: extensor carpi radialis longus; ECU: extensor carpi ulnaris, EDC: extensor digitorum communis; EDM: extensor digiti minimi; FCR: flexor carpi radialis; FCU: flexor carpi ulnaris; FDP: flexor digitorum profundus; FDS: flexor digitorum superficialis; FPL: flexor pollicis longus; PT: pronator teres. (**c**) Electrode placement with targeted method. Six forearm muscles were targeted in this study. The most prominent area of ECRL/B, which is 3 cm distal to the elbow joint, was the target area for the muscle. The upper third area between the lateral epicondyle and the midpoint of the wrist joint was the target site for EDC. Additionally, the midpoint between the lateral epicondyle and the ulnar styloid was targeted for ECU. Electrodes for FCR were located at the proximal one-third of the way between the tendon of FCR at the wrist and the medial supracondylar area of the humerus. The midforearm area between palmaris longus and FCU was the target area for FDS. Finally, FCU was targeted at the midline between the ulnar styloid process and the medial epicondyle [28]. (**d**) Electrode placement with untargeted method. Six surface electromyography (sEMG) channels were located equally around the circumference of the forearm.

We recorded sEMG data on the dominant forearm muscles from six different control sites. At each site, pairs of dry Ag/AgCl sEMG electrodes were placed around the circumference of the forearm. According to the guidelines for sEMG for the non-invasive assessment of muscles, the inter-electrode distance was set to 2 cm [27]. A ground electrode was placed on the olecranon of the dominant forearm. In the targeted methods, we placed sEMG electrodes on six specific forearm muscles, which play distinct roles in specific hand movements. The six specific forearm muscles were extensor carpi radialis longus and brevis (ECRL/B), extensor digitorum communis (EDC), extensor carpi ulnaris (ECU), flexor carpi radialis (FCR), flexor digitorum superficialis (FDS), and flexor carpi ulnaris (FCU). Each electrode was placed according to the guidelines recommended by Lee et al. to obtain the most accurate motor unit potentials of the targeted muscles (Figure 1a) [28]. The identification of the targeted muscles and accurate location of the electrodes were carried out by a physiatrist with extensive experience in localizing the muscles under investigation.

In the untargeted methods, we placed sEMG electrodes on six uniformly spaced areas of the forearm. The electrodes were arranged on the forearm at 40% of the distance from the medial epicondyle of the humerus to the styloid process of the ulnar (Figure 1b) [16]. During the recording time, the subjects sat comfortably in armchairs and rested their hands on a desk.

### 2.3. Experimental Protocol

**Figure 2:**
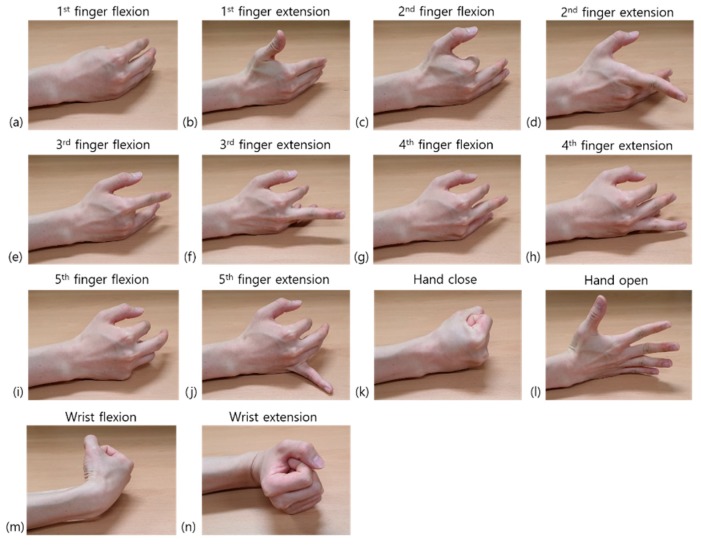
The 14 different hand movements that were used in this study. (**a**) 1st finger flexion. (**b**) 1st finger extension. (**c**) 2nd finger flexion. (**d**) 2nd finger extension. (**e**) 3rd finger flexion. (**f**) 3rd finger extension. (**g**) 4th finger flexion. (**h**) 4th finger extension. (**i**) 5th finger flexion. (**j**) 5th finger extension. (**k**) Hand closing. (**l**) Hand opening. (**m**) Wrist flexion. (**n**) Wrist extension.

Each subject performed a series of sessions comprising 14 different hand movements (Figure 2). In each session, the graphical user interface software displayed 14 different types of visual cues in serial order, which represented 14 different hand motions. These were 1st–5th finger flexion and extension, hand opening and closing, and wrist flexion and extension. Each visual cue was displayed for 5 s, and the rest interval between two adjacent cues was 3 s. Therefore, according to the visual cues, the subjects contracted muscles for 5 s and rested for 3 s. They were asked to perform exact hand movements by applying moderate and constant force. After completing a single session, subjects were allowed to take 10 min of rest to avoid muscle fatigue. A total of 10 sessions were performed for both the targeted and untargeted methods. Therefore, a total of 20 sessions per person were performed during the experiment.

After the data acquisition, we tried to compare the performance of DFDL and other classical classifiers and investigated the difference in classification accuracy according to the electrode location in each classifier. Because the main purpose of the study was to find the optimal method to classify hand gestures with a minimum number of channels, classification accuracy according to the number of channels was also analyzed. Finally, we investigated the performance of the DFDL algorithm in essential hand movements. Grasping and releasing are fundamental functions of hands. Most commercially available myoelectric prostheses offer the combination of these two movements [7]. Additionally, according to an internet survey of myoelectric prosthetic hand users, pointing with an extended index finger is the most desired additional hand movement in future prosthetic hand developments [29]. Therefore, the opening and closing of hands and second finger flexion and extension were selected, and the sEMG of these four motions were analyzed with DFDL.

### 2.4. Other Database

We evaluated our algorithm using the Non-Invasive Adaptive Prosthetics (NINAPRO) database. The NINAPRO database contains a dataset of up to 53 hand movements of various signals, such as sEMG, hand kinematics, and hand dynamics, from intact subjects and amputee patients [30]. Among these datasets, we used the NINAPRO dataset 3, which measures sEMG signals from amputees. It is a dataset that measures sEMG at a sampling rate of 2000 Hz from 11 amputees using a 12-channel-based sEMG measurement system. The disabilities of the arm, shoulder, and hand (DASH) scores of the participating amputees ranged from 1.67 to 86.67 (0–100). Experiments were repeated six times for 40 hand movements, and each hand movement was held for 5 s and rested for 3 s between the movements [31,32].

### 2.5. Acquisition Setup

To obtain the sEMG signals, a six-channel EMG acquisition board was developed for each channel via the Arduino platform with a gain of 2000x. The sEMG signals were band-pass filtered for the frequency range of 5–250 Hz, which has the most useful energy when sampled at 500 Hz using a 12-bit analog to digital converter [33]. The raw sEMG signal was transmitted to a personal computer via serial communication. The acquisition software was developed based on MATLAB (MathWorks, Natick, MA, USA).

### 2.6. EMG Signal Processing and Feature Extraction

A notch filter (59–61 Hz) was used to eliminate the power line noise of the measured sEMG signal. The window size was fixed at 200 ms (100 samples) and had an overlap of 100 ms (50 samples) to extract features from the sEMG trials [34]. To analyze the data, we divided the data into training sets that consisted of 9 randomly selected trials per task and test sets that consisted of the remaining 1 trial per task using 10-fold cross-validation. Then, we calculated the classification accuracy for each subject. We trained classifiers using the features extracted from the training set and tested the performance of the classifiers using the features extracted from the test set. In the NINAPRO dataset 3, we used 6-fold cross-validation to divide the dataset into 5 randomly selected trials per task for training and the remaining 1 trial per task for the test.

The most common features of sEMG are time-domain features (root-mean-square, maximum absolute value, zero crossing, slope sign change, etc.) and frequency-domain features (mean frequency, median frequency, peak frequency, mean power, total power, etc.) [35]. In this study, we used the 4th order wavelet packet transform coefficients feature, because it contains both time- and frequency-domain characteristics [36,37]. MATLAB was used for sEMG signal processing, feature extraction, and multiclass classification.

### 2.7. Classification

#### 2.7.1. Discriminative Feature-Oriented Dictionary Learning

Let a=Db be a linear equation, where **D** is an undetermined m × n matrix (m≪n), known as a dictionary matrix, and $a\in {\mathbb{R}}^{m}$, $b\in {\mathbb{R}}^{n}$. Signal ***a*** can be represented by vector ***b*** using the above linear equation. Typically, l1-norm minimization method is used in sparse approximation method [38,39]. Let $y\in R^{d}$ be a small window extracted from a signal, which will be referred to as a sample. In C-class classification, all data samples from class *i* (*i* can vary between 1 and *C*) compose the matrix $Y_{i}\in {\mathbb{R}}^{d\times N_{i}}$, and all complementary data samples that are not in class *i* compose the matrix ${\bar{Y}}_{i}\in {\mathbb{R}}^{d\times {\bar{N}}_{i}}$, where $N_{i}$ and ${\bar{N}}_{i}$ are the number of samples of class *i* and the complementary number of samples of *i*, respectively. Next, $D_{i}\in {\mathbb{R}}^{d\times k_{i}}$ denotes the dictionary of class *i* that is to be learned through the DFDL method, where *k* is the number of bases in the dictionary.

l0-norm of a sparse vector $s\in {\mathbb{R}}^{k}$, $\left\|s_{0}\right\|$, is the number of its non-zero elements. For a matrix **S**, the sparsity constraint $\|S_{0}\|<L$ indicates that each column of **S** contains less than *L* non-zero elements.

To train class-specific dictionaries $D_{i}$, where each $D_{i}$ can sparsely represent samples from class *i* and has poor representation of its complementary samples, the following equations are required:

(1) $$\underset{\|s_{l}\|{}_{0}\leq L_{i}}{\min}\|y_{l}-D_{i}s_{l}\|{}_{2}^{2},\;\forall l=1,2,\cdots ,N_{i}\;\mathrm{to}\;\mathrm{be}\;\mathrm{small}$$

and

(2) $$\underset{\|s_{m}\|{}_{0}\leq L_{i}}{\min}\|{\bar{y}}_{m}-D_{i}{\bar{s}}_{m}\|{}_{2}^{2},\;\forall m=1,2,\cdots ,{\bar{N}}_{i}\;\mathrm{to}\;\mathrm{be}\;\mathrm{large}$$

where $L_{i}$ handles the sparsity level. The above conditions can be simplified in the form of matrices as follows:

(3) $$\mathrm{Intra}\;\mathrm{class}\;\mathrm{differences}:\frac{1}{N_{i}}\underset{\|S_{i}\|{}_{0}\leq L_{i}}{\min}\|Y_{i}-D_{i}S_{i}\|{}_{F}^{2}\;\mathrm{small},$$

(4) $$\mathrm{Inter}\;\mathrm{class}\;\mathrm{differences}:\;\frac{1}{{\bar{N}}_{i}}\underset{\|{\bar{S}}_{i}\|{}_{0}\leq L_{i}}{\min}\|{\bar{Y}}_{i}-D_{i}{\bar{S}}_{i}\|{}_{F}^{2}\;\mathrm{large}.$$

Because only one class was considered for simplification, class index *i* was dropped. The optimized dictionary, $D^{*}$, can be calculated as follows:

(5) $$D^{*}=\underset{D}{\mathrm{argmin}}\left(\frac{1}{N}\underset{{\left\|S\right\|}_{0}\leq L}{\min}\|Y-DS\|{}_{F}^{2}-\frac{\rho }{\bar{N}}\underset{{\left\|\bar{S}\right\|}_{0}\leq L}{\min}\|\bar{Y}-D\bar{S}\|{}_{F}^{2}\right)$$

where ρ denotes a positive regularization parameter. The first term in Equation (3) minimizes the intra-class differences, whereas the second term maximizes the inter-class differences. We can find class-specific dictionaries by solving Equation (3).

The sparsity level *L* should be determined carefully. If it is too small, the dictionary might not represent intra-class information, whereas if it is too large, the dictionary might represent complementary class information. To determine *L*, the in-class sample ***Y*** was used to train the dictionary through online dictionary learning [40]:

(6) $$\left(D^{0},S^{0}\right)=\underset{D,S}{\mathrm{argmin}}\left\{\|Y-\mathrm{DS}\|{}_{F}^{2}+\lambda {\left\|S\right\|}_{1}\right\}$$

where λ represents a positive regularization parameter that controls the sparsity level. $D^{0}$ can be used as the initialization of **D** in DFDL, and $S^{0}$ can be utilized to find the value of *L* as follows:

(7) $$L\approx \frac{1}{N}\overset{N}{\underset{i=1}{\sum }}\|s_{i}^{0}\|{}_{0}.$$

The sparse codes ***s*** can be formulated via l1-norm minimization as follows:

(8) $$\hat{s}=\underset{s}{\mathrm{argmin}}\left\{\|y-D_{t}s\|{}_{2}^{2}+\gamma {\left\|s\right\|}_{1}\right\}$$

where $D_{t}=\left[D_{1},D_{2},\cdots ,D_{C}\right]$ denotes the collection of all dictionaries and γ represents a scalar constant. The identity of ***y*** is determined as follows:

(9) $$\underset{i\in \left\{1,\cdots ,c\right\}}{\mathrm{argmin}}\left\{r_{i}\left(y\right)\right\},\;where\;r_{i}\left(y\right)=\|y-D_{i}\delta_{i}\left(\hat{s}\right)\|{}_{2}$$

and $\delta_{i}\left(\hat{s}\right)$ is an element of $\hat{s}$ associated with class *i*.

With a fixed value of **D**, the optimal sparse codes $S^{*}$ and ${\bar{S}}^{*}$ can be calculated as follows:

(10) $$S^{*}=\underset{{\left\|S\right\|}_{0}\leq L}{\mathrm{argmin}}\|Y-\mathrm{DS}\|{}_{F}^{2};\;{\bar{S}}^{*}=\underset{{\left\|\bar{S}\right\|}_{0}\leq L}{\mathrm{argmin}}\|\bar{Y}-D\bar{S}\|{}_{F}^{2}.$$

These two sparse coding problems with the same dictionary **D** can be associated as follows:

(11) $${\hat{S}}^{*}=\underset{{\left\|\hat{S}\right\|}_{0}\leq L}{\mathrm{argmin}}\|\hat{Y}-D\hat{S}\|{}_{F}^{2}$$

where $\hat{Y}=\left[Y,\bar{Y}\right]$ contains all training samples and $\hat{S}=\left[S,\bar{S}\right]$. The orthogonal matching pursuit in the SPArse Modeling Software toolbox can be used to solve Equation (11) effectively [41].

For the update of the dictionary bases, $D^{*}$ can be formulated as follows using the equation ${\left\|M\right\|}_{F}^{2}=trace\left(MM^{Τ}\right)$:

(12) $$D^{*}=\underset{D}{\mathrm{argmin}}\left\{\frac{1}{N}\|Y-\mathrm{DS}\|{}_{F}^{2}-\frac{\rho }{\bar{N}}\|\bar{Y}-D\bar{S}\|{}_{F}^{2}\right\},$$

(13) $$=\underset{D}{\mathrm{argmin}}\left\{-2trace\left(PD^{Τ}\right)+trace\left(DRD^{Τ}\right)\right\}$$

where

(14) $$P=\frac{1}{N}YS^{Τ}-\frac{\rho }{\bar{N}}\bar{Y}{\bar{S}}^{Τ};\;R=\frac{1}{N}SS^{Τ}-\frac{\rho }{\bar{N}}\bar{S}{\bar{S}}^{Τ}.$$

**Figure 3:**
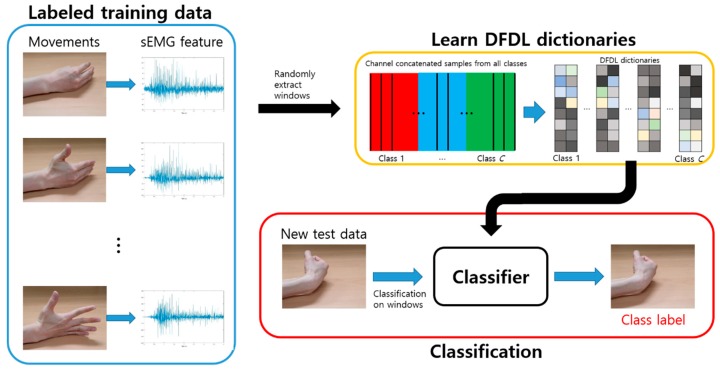
Proposed framework for sEMG classification using discriminative feature-oriented dictionary learning (DFDL).

The overall classification procedure is shown in Figure 3.

DFDL bases are trained in each class. Training windows are randomly extracted from labeled training data, which might be overlapping. The size of the windows is chosen by cross-validation. After extracting a set of windows from all classes, class-specific DFDL dictionaries and the associated classifiers are trained. After training, test signals are divided into non-overlapping windows. These windows are then classified using the previously learned DFDL model.

#### 2.7.2. Classical Classifiers

We compared the performance of DFDL with other classical classifiers. The classical classifiers used for comparison were support vector machine with linear kernel (SVM_lin), support vector machine with radial basis function kernel (SVM_rbf), LDA, naïve Bayes classifier (NB), random forests (RF), and k-nearest neighbors (KNN) [30,32]. SVM computes the hyperplanes that maximize the geometric margin, which is the distance between the two closest training samples from the hyperplane. LDA focuses on the hyperplanes, which linearly separate data by minimizing within-class scatter and maximizing between-class scatter [42]. Naïve Bayes is a classifier based on the conditional probability theory that compares the score of the test data with a predefined threshold based on the training set to calculate the classification accuracy. Random forest is a collection of decision trees and calculates the classification accuracy by combining the result of each decision tree. Finally, KNN determines a class by calculating the distance between the test data and the nearest k training data [43].

### 2.8. Statistics

As the results showed normal distribution in the Shapiro–Wilk test (*p*-value >0.05), parametric statistics were used to analyze data. In addition, repeated-measures analysis of variance (ANOVA) was conducted to compare the effects of classifiers and the number of channels. Bonferroni correction was performed after ANOVA. Subsequently, a paired t-test was performed to compare DFDL and SVM directly and to analyze the effect of targeting electrodes. All the statistical analyses were done using MATLAB. For all tests, the statistical significance was set to 0.05.

## 3. Results

### 3.1. Classification Results for the Healthy Volunteers

Among the 23 volunteers in this study, sEMG data from 22 volunteers were analyzed. Data from one volunteer were noisy and consequently excluded from the analysis. The data included sEMG signals of each different hand movement. Table 1 shows the direct comparison of classification accuracy from different classifiers using repeated-measures ANOVA.

The results showed that DFDL demonstrated the highest classification accuracy among all classifiers, and its performance was statistically significant except with respect to SVM_rbf. This trend was equally applied to both the targeted and untargeted methods. Further studies were conducted to validate the performance and characteristics of DFDL. The classification accuracy of DFDL according to the number and location of channels was compared with those of SVM_rbf.

### 3.2. Comparison between DFDL and SVM

**Figure 4:**
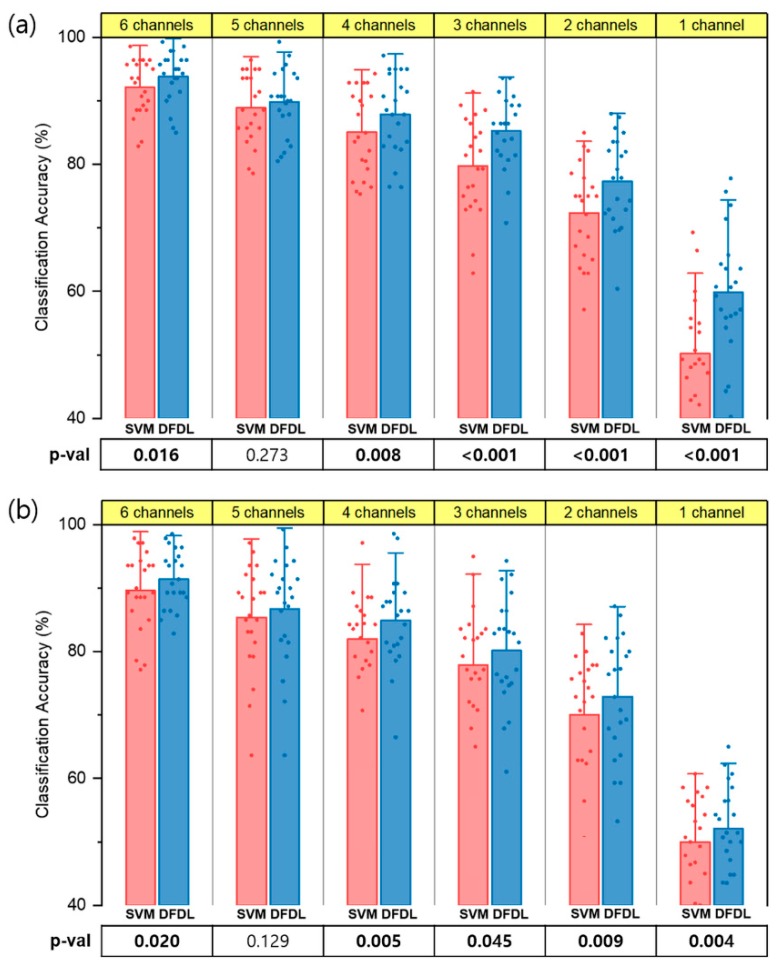
The average classification performance of DFDL and SVM_rbf for different numbers of channels. Overall, the performance of DFDL was better than that of SVM_rbf, and the gap became more obvious as the number of channels decreased. (**a**) The targeted method. (**b**) The untargeted method. Each dot indicates the classification accuracy of each subject. The error bar represents the standard deviation. Bold values indicate significant differences.

Figure 4 shows the performance of DFDL and SVM_rbf with both the targeted and untargeted methods for myoelectric pattern recognition. The sEMG data of 14 different hand movements were analyzed according to the number of electrodes. First, all the combinations of channels were tested, and the classification accuracy of each channel subset was calculated. Next, the best channel subsets in the range of 1–6 were selected. Subsequently, the mean classification accuracy of each classifier was compared. In both the targeted and untargeted methods, the classification accuracy of DFDL outperformed SVM_rbf in most of channel subsets on paired t-tests (*p* < 0.05). There was no exceptional difference between the two classifiers when using 5 channels (*p* = 0.273 in the targeted method and *p* = 0.129 in the untargeted method). Furthermore, as the number of channels decreased, the performance difference between two classifiers became evident, particularly in the targeted method.

### 3.3. Effects of Number and Location of Electrodes

**Figure 5:**
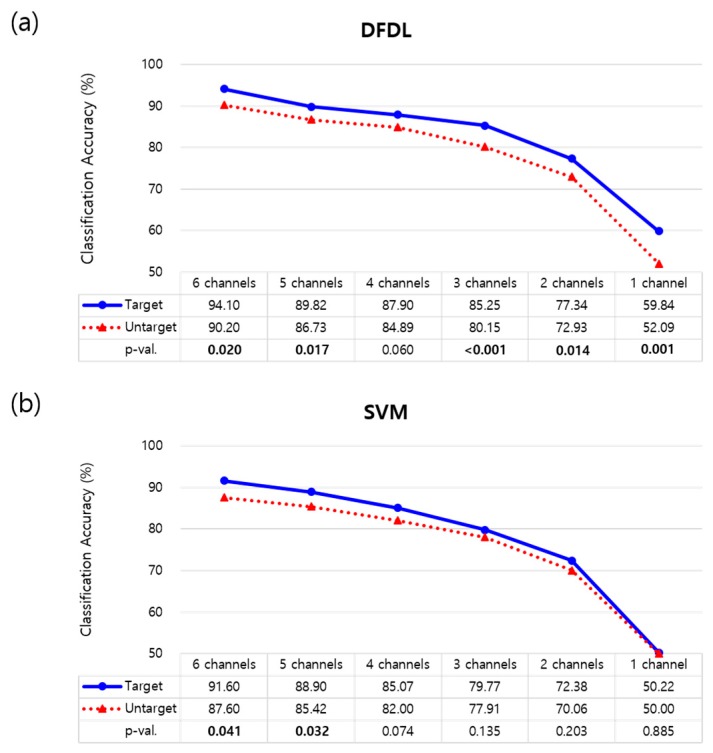
Comparison of the targeted and untargeted method. (**a**) The targeted method showed higher classification accuracy than the untargeted method with statistical significance in every number of channels except 4 channels in DFDL. (**b**) However, the targeted method was superior to the untargeted method only when using 5–6 channels in SVM. Bold values indicate significant differences.

The optimal channel subsets were further investigated, and the effects of the targeted method were evaluated. The classification accuracy of each classifier according to the number of channels is shown in Figure 5. When classifying sEMG data using DFDL, targeting electrodes on proper sites showed better performance than not targeting electrodes in almost all channel subsets with statistical significance (*p* < 0.05) (Figure 5a). Although not statistically significant, the targeted method showed higher performance than the untargeted method when recording from 4 channels (*p* = 0.060). However, when the data were analyzed using SVM_rbf, the targeted method was more efficient than the untargeted method when using 5–6 channels. There was no statistical difference according to the targeted method when using 1–4 channel subsets in SVM_rbf (Figure 5b).

Next, a trend of decreasing accuracy was found when the number of channel subsets decreased in both DFDL and SVM_rbf (Figure 5). A significant decrease in the classification accuracy was observed when using only 1 channel. However, while comparing the classification accuracy of each channel subset, DFDL appeared to be more effective than SVM_rbf in terms of reducing the number of channels. In the targeted method with DFDL, there was no statistical difference between performance using 3 channels and 4 channels on repeated-measures ANOVA (*p* = 0.124). In addition, the classification rate determined using 4 channels was as high as that using 5 channels in both the targeted (*p* = 0.116) and untargeted methods (*p* = 0.551). These results suggest that classification accuracy can be maximized using DFDL, even with a smaller number of channels. In contrast, the classification accuracy of SVM_rbf decreased significantly when the number of channels decreased in the all the channel subsets (*p* < 0.05).

While analyzing the optimal location of channel subsets in the targeted method, the FCU muscle was found to be the most informative when using only 1 channel, followed by FDS and EDC, similar to the results of a previous study [16]. However, when combining channels, the combinations of ECR and FDS (2 channels) and ECR, FDS, and FCU (3 channels) showed the highest accuracy. These results suggest that combining channels located on the flexor and extensor muscles appropriately increases the classification accuracy. On 4 channels, combining ECR, FDS, FCU, and ECU demonstrated the highest accuracy, and adding EDC to the subsets was the best combination for the 5 channels. Although the location of optimal channels between the subjects was not always the same, the overall tendencies described above were observed in both DFDL and SVM_rbf.

### 3.4. Classification Results of Each Class

**Figure 6:**
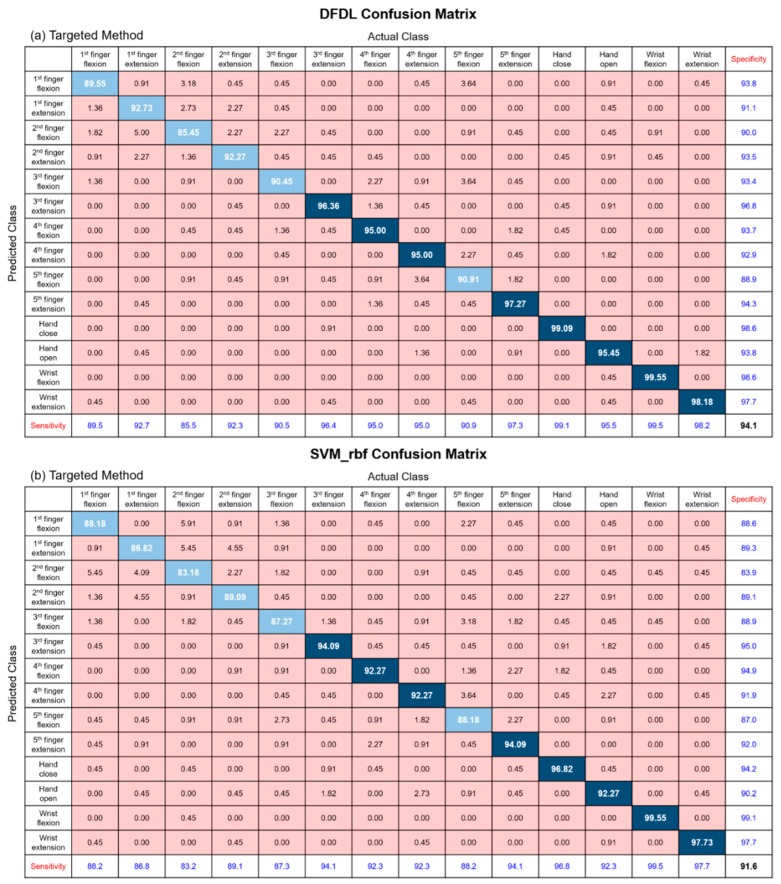
The classification results of each class using the targeted method represented by a confusion matrix. (**a**) DFDL with the targeted method. (**b**) SVM_rbf with the targeted method. The diagonal elements represent the classification accuracy for which the predicted class is equal to the actual class, while the off-diagonal elements indicate those that are misclassified. Sensitivities and specificities for each class are calculated.

**Figure 7:**
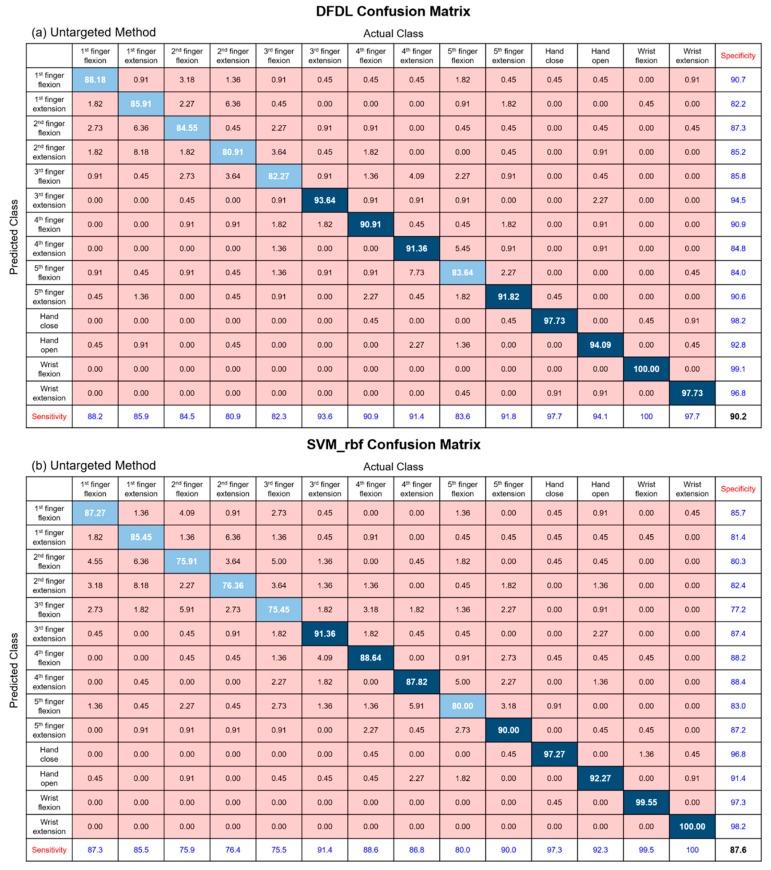
The classification results of each class using the untargeted method represented by a confusion matrix. (**a**) DFDL with the untargeted method. (**b**) SVM_rbf with the untargeted method. The diagonal elements represent the classification accuracy for which the predicted class is equal to the actual class, while the off-diagonal elements indicate those that are misclassified. Sensitivities and specificities for each class are calculated.

In order to determine whether certain hand gestures affect the classification accuracy of classifiers, we analyzed the sensitivity of each class using confusion matrices. Figure 6 and Figure 7 represent the results of each confusion matrix according to classifiers and location of the electrodes. According to the results, the accuracy of the 1st finger and 2nd finger movements showed lower sensitivities when compared with other movements. Additionally, the classification of the 3rd and 5th finger flexion movements showed relatively low sensitivities. The results of the confusion matrices showed similar tendency regardless of the classifiers and electrode locations. This is probably due to the relationship between the anatomical features of the forearm muscles and the hand movements performed in the experiment. First of all, the flexion and extension of the 1st finger are mainly controlled by the flexor pollicis longus and extensor pollicis longus muscles, and the 2nd finger extension is controlled mostly by extensor indicis proprius. The problem is that these muscles are distal forearm muscles, and therefore, it is almost impossible to record sEMG signals with this experimental paradigm, which places electrodes on the proximal forearm. Next, flexion of the 2nd–5th fingers are controlled by both the FDS and flexor digitorum profundus (FDP) muscles. While FDS acts only as a flexor for the proximal interphalangeal joints, FDP is a flexor for both metacarpophalangeal joints and every interphalangeal joint. Therefore, FDP is also mainly involved in the flexion of the 2nd–5th finger [44]. However, because this muscle is deep muscle lying under FDS, it is difficult to obtain accurate sEMG signals, and this limitation might affect the experimental results. On the other hand, sensitivities of gross hand movements, such as hand opening and closing and wrist flexion and extension, were higher than those of dexterous movements.

### 3.5. Classification Results for Amputees

As myoelectric prostheses are applied to patients having undergone amputation, the NINAPRO dataset 3 was used to validate the proposed framework, including DFDL. Table 2 shows the classification accuracy of the database using various classification methods. Among the classifiers, the classification rate using DFDL was the highest and was statistically significant except with respect to SVM_rbf. The overall classification accuracy of the NINAPRO dataset 3 was lower than that of the healthy volunteers. This was probably because forearm muscle atrophy might occur and actual visual feedback cannot be obtained, whether the intended hand movements were correct or not. Additionally, the number of hand movements to classify was much higher in that dataset than in our study, even though the NINAPRO dataset 3 was recorded with more channels. These results suggest that DFDL can be applied to classify the sEMG data of amputee subjects and shows a higher performance rate than other classifiers.

### 3.6. Classification Accuracy of Fewer Hand Movements

Eventually, we tried to reduce the number of hand movements to the most essential everyday movements in life, which were hand opening and closing and second finger flexion and extension. By reducing the number of classifications to four, we expected to achieve a higher classification rate with a small number of channels. We tried to determine if high accuracy could be obtained with a small number of channels, which could then be applied to real-world myoelectric prostheses.

**Figure 8:**
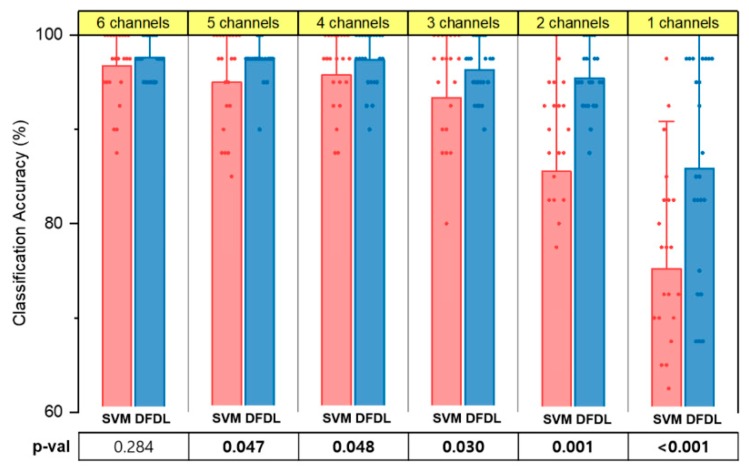
The classification performance of DFDL and SVM_rbf for four essential hand movements using the targeted method. The performance of DFDL was superior to SVM_rbf in every number of channel subsets. Moreover, a decrease in performance was not observed until the number of channels was reduced to 2 in DFDL. Each dot indicates the classification accuracy of each subject. The error bar represents the standard deviation. Bold values indicate significant differences.

In accordance with the previous analysis, every combination of channels was evaluated, and the best subsets in the range of 1–6 channels were selected. As the targeted method was superior to the untargeted method, we compared DFDL and SVM_rbf using the targeted method. Similar to the study of 14 hand movements, DFDL surpassed SVM with statistical significance in most of the channel subsets. Moreover, the results showed that the difference between the two classifiers was more evident when the number of channels decreased (Figure 8). In particular, the accuracy of DFDL was not compromised, even with a small number of channels. While using 2 channels, the accuracy was more than 95%, which was not statistically significant when compared with 6 channels on repeated-measures ANOVA (*p* = 0.334). Stated differently, only 2 channels are sufficient to demonstrate a good performance when classifying four hand movements in DFDL. However, in the case of SVM_rbf, at least four channels were required to show similar performance to 6 channels.

## 4. Discussion

In this study, we proposed DFDL as an efficient algorithm for myoelectric pattern recognition. To our knowledge, this is the first study that has applied this algorithm for myoelectric pattern recognition. The proposed method showed excellent performance while classifying the sEMG data for different hand movements compared with other methods, particularly for a small number of recording channels. Although it appeared to be only a few percent increases, the performance of the DFDL was superior to other classifiers under the controlled conditions, and this was proven using statistical methods. Furthermore, after reducing the number of hand movements that needed to be classified, DFDL showed much better performance than SVM, which was suitable enough for application in real prostheses. In addition, we identified several advantages of the targeted approach. Though the targeted approach might be expected to produce better performance, no study before this one has proven the effectiveness of this method. In the study, we proved that placing electrodes on functionally relevant muscles could enhance the classification accuracy, especially when combined with the DFDL algorithm. This study is therefore significant as it suggests an optimal method for myoelectric pattern recognition, which can achieve greater classification accuracy even with a small number of channels. Furthermore, this study is statistically more powerful than previous studies, as more subjects were analyzed with more classifications [15,16,45]. Subsequently, we tried to analyze the sEMG of individual finger movements for dexterous prosthetic control, which is relatively weak and difficult to classify as compared with typical hand movements.

DFDL constructs a dictionary by dividing each class data into small windows in a training dataset and obtaining sparse codes. Therefore, we achieved better results by emphasizing inter-class variation and encouraging intra-class similarities of the extracted features compared with other classification algorithms [20]. As DFDL constitutes a class-specific dictionary, the results show that the loss of information caused by reducing the number of channels had better compensation than other algorithms. Additionally, DFDL can be significantly more accurate when sEMG is measured from selected target muscles due to the class-specific dictionary (Figure 5). sEMG signals obtained from amputee patients are weak when compared with those of healthy subjects, and it is difficult to extract the features of each class [46]. Previous sEMG studies analyzed a small number of trials and used a large number of channels [16,32]. However, the best results can be obtained using the proposed method even when information is inadequate, such as in the cases of sEMG from a reduced number of channels or amputee patients.

The surface electrode is advantageous in that it is noninvasive and easy to apply as compared with the needle electrode. Consequently, almost all studies on prosthetic control use sEMG data [16]. The surface electrode can be either placed on the targeted muscles or arrayed symmetrically around the forearm. Because it is easy to arrange the electrodes equally and it can be performed by a non-medical practitioner, many studies prefer to use the untargeted method. However, the targeted method has a few advantages over the untargeted method. By placing electrodes on targeted sites, it is possible to record consistently and minimize the differences of inter-subject EMG data. Furthermore, because sEMG data are recorded on a focal muscle, crosstalk is relatively small, and a more accurate signal can be obtained [47]. However, there have been a few in-depth studies about the effects of electrode location on the classification accuracy of myoelectric prostheses. Only one study directly compared the two methods to determine which method is superior. Farrell et al. classified 12 hand movements with eight channels using LDA classifiers [16]. However, no significant difference between the two methods was reported in that study.

A possible explanation for why the results of Farrell’s study and our study are contradictory is that different hand movements were analyzed. Farrell et al. tried to classify distinct hand postures, such as wrist flexion, pronation, radial deviation, and lateral prehension. The myoelectric pattern recognition of such gross movements is relatively easy, because the sEMG data is relatively large and clear. Additionally, when analyzing the accuracy per class, the accuracy and sensitivity of gross hand movements, such as hand opening and closing, were higher than those of dexterous movements in this study. Therefore, it might be possible to classify the hand movements sufficiently even with an untargeted sEMG signal. However, we classified dexterous movements. Classifying each individual movement is more challenging, because they have a large degree of freedom, and the movements are generally small and delicate [26]. Therefore, the targeted method is advantageous for analyzing dexterous movements in that it can obtain more accurate signals from each muscle than the untargeted method. In addition, the previous study selected the supinator muscle as one of the target muscles. The problem with this selection is that the majority of the supinator muscle is located inside the deep layer of the posterior forearm, and the extensor muscles cover the supinator muscle [44]. Therefore, it is difficult to record accurate signals of the supinator muscle without interference from signals of other overlying muscles.

This study reveals the effectiveness of the targeted method in classifying dexterous movements. In particular, when combined with DFDL, high classification accuracy can be achieved with a minimal number of channels. In most of the cases in this study, DFDL showed better classification accuracy than SVM, and therefore, it can be used as a new and efficient classifier in gesture classification of hand prostheses. Moreover, the algorithm is expected to be a good candidate for application in other human–computer interfaces (HCI) that need to classify different motor tasks based on sEMG. For instance, DFDL might be suitable for driving an electric-powered wheelchair or for facial gesture recognition devices. According to previous studies, the number of channels applied to these HCI was relatively small [48,49,50]. Therefore, DFDL might improve the performance of such devices, which use conventional classifiers.

The proposed framework can be further developed in the future. Although the algorithm was not tested during online control, dictionary learning has a fast testing time [20,51]. Therefore, DFDL could be used in real-time application. Additionally, as DFDL is an appropriate algorithm for reducing the number of channels, it will not only improve a user’s convenience, but also reduce the computational power and complexity of the hardware. Moreover, the performance of DFDL can be improved by locating electrodes on other specific muscles. As this experiment was designed to apply prostheses for proximal transradial amputation patients, we did not place electrodes on distal forearm muscles, such as extensor pollicis longus or extensor indicis proprius. These muscles directly control the thumb and index fingers but end in the distal third of the forearm. Considering the condition of an amputated forearm, it is expected that higher classification accuracy would be acquired if we additionally targeted these relevant muscles. Finally, combining deep learning approaches, such as convolutional neural network or recurrent neural network, to train sEMG signals will be useful for future neuroprosthesis control. Publications discussing deep learning for biological signal processes including sEMG have been increasing significantly. According to recent studies, deep learning methods showed high accuracy and outperform conventional machine learning techniques with respect to gesture recognition [13,52]. Because lots of varied sEMG data are produced in the clinical setting, the convergence of the proposed method and the deep learning approach could be the start of a paradigm shift in myoelectric signal classification.

## 5. Conclusions

The primary purpose of this study was to investigate the optimal method for sEMG classification with a minimal number of channels. We concluded that combining the DFDL algorithm with targeted electrodes is the most effective method when the number of channels is small. This is the first study to utilize DFDL for myoelectric signal classification. This study is also meaningful, because it demonstrated the significant effects of the targeted method for the first time. The targeted approach using DFDL is particularly effective when classifying dexterous movements with a small number of channels. As a result, DFDL with the targeted method can be used to minimize the number of channels, and it is expected to lower the complexity and processing time of hardware. In future research, we will apply the proposed method and deep learning algorithms in real time to myoelectric prostheses.

## Figures and Tables

**Table 1:** Average Classification Accuracies for Healthy Volunteers.

	SVM_rbf	SVM_lin	LDA	KNN	NB	RF	DFDL
Targeted Method							
Acc (%)	91.6	90.6	69.9	90.4	73.2	62.1	94.1
*p*-val	0.343	**0.018**	**0.000**	**0.001**	**0.000**	**0.000**	
Untargeted Methods							
Acc (%)	87.6	86.5	65.7	86.0	66.0	60.4	90.2
*p*-val	0.414	**0.047**	**0.000**	**0.020**	**0.000**	**0.000**	

SVM_rbf = support vector machine using radial basis function, SVM_lin = support vector machine using linear kernel function, LDA = linear discriminant analysis, KNN = k-nearest neighbor, RF = random forest, DFDL = discriminative feature-oriented dictionary learning, Acc = accuracy, *p*-val = *p*-value. Bold values indicate significant differences.

**Table 2:** Average Classification Accuracies for Amputee Subjects.

	SVM_rbf	SVM_lin	LDA	KNN	NB	RF	DFDL
Acc (%)	62.3	60.7	35.8	55.5	35.6	24.6	65.1
*p*-val	0.736	0.292	**0.000**	**0.002**	**0.000**	**0.000**	

## Abbreviations

The following abbreviations are used in this manuscript:

sEMG	Surface electromyography
DFDL	Discriminative feature-oriented dictionary learning
LDA	Linear discriminant analysis
SVM	Support vector machine
K-SVD	K-singular value decomposition
EEG	Electroencephalogram
LC-KSVD	Label consistent K-SVD
ECRL/B	Extensor carpi radialis longus and brevis
EDC	Extensor digitorum communis
ECU	Extensor carpi ulnaris
FCR	Flexor carpi radialis
FDS	Flexor digitorum superficialis
FDP	Flexor digitorum profundus
FCU	Flexor carpi ulnaris
NINAPRO	Non-Invasive Adaptive Prosthetics
DASH	Disabilities of the arm, shoulder, and hand
SVM_lin	Support vector machine with linear kernel
SVM_rbf	Support vector machine with radial basis function kernel
NB	Naïve Bayes classifier
RF	Random forests
KNN	k-nearest Neighbors
ANOVA	Analysis of variance
